# Numerical processing efficiency improved in children using mental abacus: ERP evidence utilizing a numerical Stroop task

**DOI:** 10.3389/fnhum.2015.00245

**Published:** 2015-05-19

**Authors:** Yuan Yao, Fenglei Du, Chunjie Wang, Yuqiu Liu, Jian Weng, Feiyan Chen

**Affiliations:** ^1^Department of Psychology and Behavioral Sciences, Zhejiang UniversityHangzhou, China; ^2^Bio-X Laboratory, Department of Physics, Zhejiang UniversityHangzhou, China

**Keywords:** mental abacus, numerical stroop paradigm, numerical processing, interference, facilitation

## Abstract

This study examined whether long-term abacus-based mental calculation (AMC) training improved numerical processing efficiency and at what stage of information processing the effect appeard. Thirty-three children participated in the study and were randomly assigned to two groups at primary school entry, matched for age, gender and IQ. All children went through the same curriculum except that the abacus group received a 2-h/per week AMC training, while the control group did traditional numerical practice for a similar amount of time. After a 2-year training, they were tested with a numerical Stroop task. Electroencephalographic (EEG) and event related potential (ERP) recording techniques were used to monitor the temporal dynamics during the task. Children were required to determine the numerical magnitude (NC) (NC task) or the physical size (PC task) of two numbers presented simultaneously. In the NC task, the AMC group showed faster response times but similar accuracy compared to the control group. In the PC task, the two groups exhibited the same speed and accuracy. The saliency of numerical information relative to physical information was greater in AMC group. With regards to ERP results, the AMC group displayed congruity effects both in the earlier (N1) and later (N2 and LPC (late positive component) time domain, while the control group only displayed congruity effects for LPC. In the left parietal region, LPC amplitudes were larger for the AMC than the control group. Individual differences for LPC amplitudes over left parietal area showed a positive correlation with RTs in the NC task in both congruent and neutral conditions. After controlling for the N2 amplitude, this correlation also became significant in the incongruent condition. Our results suggest that AMC training can strengthen the relationship between symbolic representation and numerical magnitude so that numerical information processing becomes quicker and automatic in AMC children.

## Introduction

Many studies using a variety of techniques, such as single-cell recording, neuroimaging, and electroencephalographic(EEG) recording have provided evidence for the important role numerical cognition plays in the domain of higher cognition (Nieder and Dehaene, 2009). Although animals can differentiate small precise quantities and represent the approximate magnitude of large sets, only human beings appear able to represent and manipulate large, exact numerical quantities (Feigenson et al., 2004). This human capacity may constitute the basis for higher arithmetical abilities (Dehaene et al., 1999; Frank et al., 2008).

In addition to the typical method gained from formal school education, there is a unique strategy based on the abacus, a traditional calculator in ancient Asia, especially in China. Children exposed to Abacus-based Mental Calculation (AMC) training can solve complex calculation problems with unusually fast speed and high accuracy (Hatano et al., 1977; Stigler, 1984). Experts acquire this capacity through a particular algorithm and after long-term training (Hatano et al., 1977).

Initially, children learn to calculate on a real abacus, with both hands simultaneously. Later, when they are getting familiar with the operation, they are instructed to simulate the abacus-calculation process in their minds with actual finger movements in the air. Finally, they can calculate via the imaginary abacus without moving fingers, as if manipulating a “mental abacus” (MA; Hatano et al., 1977; Stigler, 1984; Hanakawa et al., 2003; Chen et al., 2006; Frank and Barner, 2012).

However, to our knowledge, no study has examined the temporal course of numerical processing in AMC experts using EEG and ERP technique. Considering the extraordinary performance on number calculation by these experts, it is intriguing to examine which cognitive processing stage during numerical processing is modulated by AMC training? What is the concrete ERP component related to access to numerical representation and magnitude comparison? Which component is more related to the response organization and execution process? Since no previous studies have reported the temporal dynamics of numerical processing in AMC experts, our study was exploratory in nature.

To study the numerical processing performance, one of the most frequently used tasks is the numerical Stroop paradigm (NSP), which examines the efficiency of extracting numerical information both intentionally and automatically (Wang et al., 2013). In this task, subjects compare two numbers presented simultaneously either for numerical magnitude or physical size (Besner and Coltheart, 1979; Henik and Tzelgov, 1982). The two dimensions are either congruent or incongruent with each other, which corresponds to the Congruent and Incongruent conditions in this study. Additionally, there is a third relationship between the two dimensions where one of the dimensions was kept the same value while the other dimension varies, which corresponds to the Neutral condition.

In the comparison of numerical magnitude (NC) or physical size (PC), adults and older children, always exhibit congruity effects (Rubinsten et al., 2002; Szűcs and Soltész, 2007; Soltész et al., 2011). That is, congruence of the numerical and physical information influences reaction times (RTs), such that subjects usually exhibit shorter RTs in the congruent than in the neutral condition (the facilitation effect), and longer RTs in the incongruent than in the neutral condition (the interference effect) (Szűcs and Soltész, 2007). Both the facilitation and interference effects suggest that task-irrelevant dimensions are automatically processed (Logan, 1980). Therefore, the NSP has been widely used to examine whether the numerical information processing is automatic (Tzelgov et al., 1992; Girelli et al., 2000; Rubinsten et al., 2002; Szűcs and Soltész, 2007; Rousselle and Noël, 2008). Besides, the interference effect could reflect the ability of inhibiting irrelevant and conflicting information (Rubinsten et al., 2002), which is part of the executive function (Miyake et al., 2000; Huizinga et al., 2006). Thus, NSP task may involve a combination of extracting numerical information and monitoring and resolving conflict that is typical in Stroop paradigm.

In parallel with the behavioral responses, we predicted that three event-related potential (ERP) components would reflect the numerical magnitude, conflict detection and response organization processes. First, the numerical magnitude extracting process could be related to the N1 component. In studies using number compare paradigm (compare numerical value with “5”), N1 displayed a distance effect that reflected the efficiency of accessing numerical magnitude (Dehaene, 1996; Temple and Posner, 1998; Heine et al., 2010).

Second, the ERP components which are related to conflict detection and response organization usually appear late in the time course. Previous researchers found two components, a frontal-central medial negative component (N2) and a widely-distributed late positive complex (LPC), related to the congruence effect both in the classic Stroop and numerical Stroop paradigm (Liotti et al., 2000; Szűcs and Soltész, 2007). This indicated that N2 and LPC are not the specific components related to number processing but reflect more general inhibition and organization processes.

N2 is mainly distributed in the frontal central area, with a putative source in caudal anterior cingulate cortex (ACC; van Veen and Carter, 2002a). One of the main functions of ACC is detecting conflict during information processing, and alerting higher systems involved in top-down control to resolve conflict. ACC is usually activated before reaction during correct conflict trials, which is reflected in the amplitudes of N2 component located in frontocentral area (Van Veen and Carter, 2002b).

LPC is a late positive-going ERP component, generally found to be largest over parietal scalp sites, beginning around 400–500 ms after the onset of a stimulus and lasting for a few hundred milliseconds. LPC sometimes has been referred to as a late part of the P3b (Finnigan et al., 2002), which presumably originates from activation in the parietal and temporal regions (Polich, 2007). Verleger et al. (2005) proposed that the P3b component reflected the process between perception and response initiation. In detail, LPC is related to monitoring the stimulus-classification process and whether this process could be appropriately translated into action (Verleger et al., 2005). In other words, the LPC can reflect the response organization process.

In summary, the current study attempted to explore the difference in time course of numerical processing between children who practiced AMC and their peers. Our basic assumption was that the AMC children would gain superior numerical processing efficiency compared to their peers. In terms of ERP results, we predicted there would be three main components related to the cognitive processing under the NSP paradigm: N1, N2 and LPC. The N1 should be related to accessing numerical information, while the N2 and LPC should be related to a more general conflict monitoring and executive function. If there were differences in N1 component between the two groups, we would conclude that the AMC training modulates the process of extracting numerical information. If there were differences in N2 or LPC, then it is possible that the AMC training influences conflict detection and response organization.

## Methods

### Subjects

Children were randomly assigned to two groups (AMC, Control) at primary school entry, matched for age, gender and IQ. The AMC group had received 1.5 years AMC training (2 h/per week), while the control group had no experience of physical abacus or mental abacus at all. Instead, they went through the same curriculum at school and performed the same math homework as the AMC group, but using the traditional method. At the end of grade two, a total of 33 students participated in the experiment. Fourteen students were from AMC group (age: 8.07 ± 0.43) and 19 students were from control group (age: 7.90 ± 0.61). Three subjects from the control group were excluded from the ERP analysis due to too much artifacts. All the subjects were right-handed and their visions were normal or corrected to normal. Exclusion criteria included history of neurological disease and current use of psychotropic medications or stimulants other than caffeine. The study was reviewed and approved by the institutional review board of Zhejiang University. All participants and their guardians provided their written informed consent at the beginning of the experiment according to the 1964 Declaration of Helsinki.

### Pre IQ Tests

Children’s IQs were measured before the AMC training started by using the Chinese version of the Raven’s Standardized Reasoning Test (Zhang and Wang, 1985), in order to match the intelligence in the two groups.

### Stimuli and Equipment

Experimental stimuli were presented in white on a dark background of a 17-inch CRT monitor, with a refreshing rate of 80 Hz. Twelve Arabic number pairs were chosen as the stimuli (2–7, 3–8, 7–2, 8–3, 2–3, 7–8, 3–2, 8–7, 2–2, 3–3, 7–7, 8–8). Physically small and large sized stimuli had a font-size of 40 and 50 respectively. Stimuli in the neutral condition of the NC task (the same physical size) had a medium font-size of 45. In the neutral condition, we kept one dimension the same value (same physical size or same numerical value) while the other dimension different. In NC task, the same physical value won’t influence subjects” determination on numerical value, either in the facilitation or interference way. Thus, we used the font-size 45 which is an eclectic between 40 and 50. The visual angles of stimuli were: Arial font-size 40: height 1.71°, width 0.97°; Arial font-size 45: height 1.82°, width 1.14°; and Arial font-size 50: height 2.00°, width 1.31°. Stimuli were presented using E-Prime (version 1.2) with a display mode of 800 × 600 pixels.

### Procedure

Each trial began with a fixation symbol “+” shown for 500 ms. Then, a pair of stimuli were shown for maximum 2 s, or until the subject made a response. The stimuli were followed by a pause of 300~500 ms. There were two experimental tasks (task condition), the NC and PC task. In the NC task, subjects decided which item of the pair was numerically larger; in the PC task, subjects decided which item was physically larger. Subjects gave their response by pressing a button on the side (left or right) where they detected the larger item. The relationship between numerical and physical dimensions of the number pair could be neutral, congruent or incongruent (congruency condition, as shown in Table 1). Each congruency condition consisted one third of all experimental trials.

**Table 1:** **Examples of stimulus pairs used in the experiment**.

	Neutral	Congruent	Incongruent
NC task	2 7	2 7	2 7
PC task	2 2	2 7	2 7

We adopted a 3(congruency) × 2(task) × 2(group) mixed design. The congruency and task were withine-subjects factors and group was a between-subject factor. There were 12 practice trials and 180 experimental trials (in two blocks) for each task. The order of the two tasks were designed as ABBA or BAAB (A referred to the NC task and B refer red to the PC task). Half of the subjects participated the ABBA design and the other half participated the BAAB design. Number pairs with three congruency conditions appeared in random order. So there were 60 trials for each condition. Short breaks were offered between blocks.

### Electrophysiological Recordings

Participants were tested individually in a soundproof and electrically shielded recording booth. They were asked to fixate their gaze at the center of the monitor as much as possible, to minimize movement of eyes and facial muscles, and to keep their body (especially their head) as still as possible during the presentation. Continuous EEG was recorded using the Neuroscan Synamps2 system at a sampling rate of 1000 Hz and analog-filtered DC-100 Hz. Quick-Cap 64 electrode (Ag/AgCl) sites were placed according to an International modified 10–20 system montage (Nuwer et al., 1999). Additionally, four bipolar electrooculogram (EOG) signals to monitor eye movements were set. Impedances were kept below 10 kΩ for all electrodes. All scalp electrodes, as well as the EOG signals, were referenced to the left mastoid (M1) during recording. All scalp electrodes were grounded at a point midway between Fpz and Fz.

### EEG Data Analysis

Off-line analysis was performed using the Neuroscan 4.3.3 system. EEG were re-referenced to common average and 0.5 Hz high-pass filtered. We rejected EOG interference using the regression method in Neuroscan system and rejected artifacts with the standard of ±150 μV. After artifact rejection, an average of 39 trials were left for each condition per subject, with conditions not being statistically different from each other (Keil et al., 2014). Epochs were extracted from continuously recorded EEG relative to the onset of number pairs, 200 ms before and 1000 ms after the stimuli. The mean voltage of a 200 ms segments preceding stimuli were subtracted as the baseline. Before statistical analysis, a 20 Hz (24 db/octave) low-pass filter was applied.

Our main interests concerned the facilitation and interference effects and contrasts between AMC and the control group. Inspection of the grand mean ERP waveforms indicated that there were three time windows of divergence: N1 (170~270 ms), N2 (370~470 ms) and LPC/LNC (600~800 ms) in the medial frontal and parietal regions. Thus we chose the FZ, PZ, P5 and P6 electrods in the three time windows to analyze the mean amplitudes of ERP components preceding stimuli onset (determined by Neuroscan 4.3.3 standard ERP-analysis software, with the Areareport function).

In order to locate the sources of EEG signals, we also used the EEGLAB (version 11.0.3.1b) (Delorme and Makeig, 2004), a freely available open source software toolbox (Swartz Center for Computational Neurosciences, La Jolla, CA)^1^ running under Matlab (MathWorks, Inc, Natick, MA). After preprocessing and ICA running, an equivalent current dipole model for each brain activity component map using a MNI head model (Montreal Neurological Institute) was computed. The MNI is a spherical head model co-registered to a mean brain image in the DIPFIT toolbox (Oostenveld and Oostendorp, 2002; available from sccn.ucsd.edu/eeglab/dipfit.html). Components with equivalent dipoles located outside of the model brain volume were excluded. After pre-computing, we used the PCA method (K-means algorithm) to perform components clustering across subjects and conditions. Dipole model residual variances above 15% were omitted from clustering and the final number of clusters was set at 10. Together, the 13 clusters comprised 464 ICs drawn from all 30 subjects and 3 congruence conditions, with 25 ICs as outliers.

## Results

Data were analyzed using SPSS 16.0. All the resulting *p*-values were corrected by Greenhouse-Geisser adjustments. Only the statistical significant values were reported.

### Behavioral Results

The analysis of Raven IQ scores revealed no difference between the two groups (*t* = 1.226, *p* = 0.229, Cohen’s *d* = 0.420).

Median reaction times of every condition per subject were calculated after excluding trials with incorrect responses. The means of median RTs and error rates for each experimental condition are shown in Table 2. Moreover, there were no trade-off effect between RT and accuracy in each condition.

**Table 2:** **Means of median reaction times (RTs) and error rates**.

		PC Task		NC Task	
		RT (ms)	Error rate (%)	RTs	Error rate (%)
AMC group	congruent	628 (95)	3.8	626 (94)	3.7
	neutral	629 (81)	3.7	665 (91)	6.0
	incongruent	738 (126)	12.5	759 (106)	12.3
Control group	congruent	693 (92)	3.1	837 (181)	5
	neutral	679 (85)	1.8	911 (161)	6.0
	incongruent	774 (129)	7.5	988 (168)	14.8

*Note: Standard deviations for RTs are in parentheses*.

#### ANOVA for RTs

A 2 (Task: NC or PC) × 3 (Condition: congruent, neutral or incongruent) × 2 (Group: AMC group or the control group) repeated measures ANOVA was carried on median RTs, yielding main effets of Task (*F*_(1,31)_ = 32.87, *p* < 0.001), Congruency (*F*_(2,62)_ = 149.97, *p* < 0.001) and Group (*F*_(1,31)_ = 13.55, *p* = 0.001). There were interactions between Task and Group (*F*_(1,31)_ = 22.45, *p* < 0.001), as well as between Congruency and Task (*F*_(2,62)_ = 8.42, *p* = 0.001).

**Figure 1:**
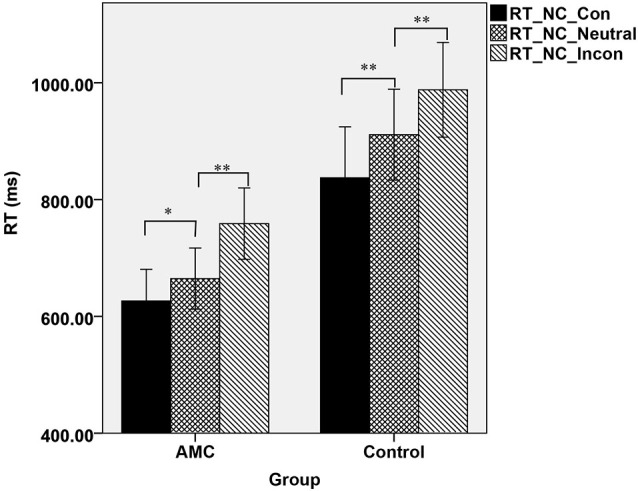
**The mean reaction times of the two groups in three congruency conditions in NC task**.

Furthermore, a simple effect analysis revealed that in NC task, there were main effects of Group (*F*_(1,31)_ = 21.64, *p* < 0.001) and Congruency (*F*_(2,62)_ = 84.15, *p* < 0.001). The AMC subjects had shorter RTs than the control group. The RT in the incongruent condition was longer than that in the neutral condition and in turn longer than that in the congruent condition. Both the AMC and the control groups had significant facilitation effects and interference effects (AMC: *p* = 0.03, *p* < 0.001; control: *p* < 0.001, *p* < 0.001). No interaction effect occurred between congrueny and group. The mean RTs of the two groups in NC task were shown in Figure 1.

**Figure 2:**
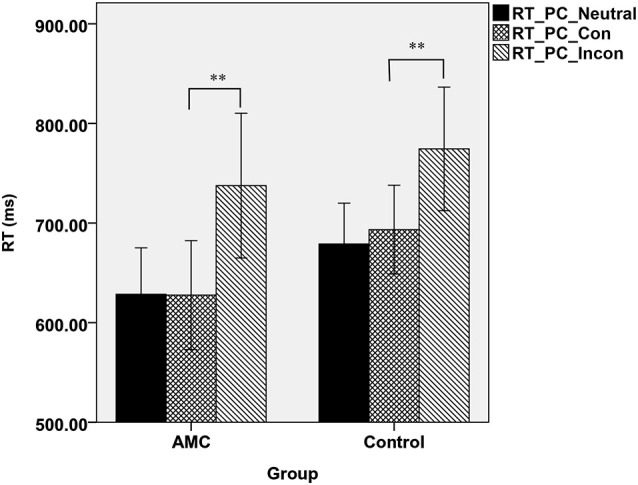
**The mean reaction times of the two groups in three congruency conditions in PC task**.

Similarly, a simple effect analysis revealed that in the PC task, only a main effect of Congruency was found (*F*_(2,62)_ = 59.98, *p* < 0.001). Both of the two groups showed interference effects (both *p* < 0.001), but neither of them showed faciliation effects. The mean RTs of the two groups in PC task were shown in Figure 2.

#### ANOVA for Accuracy

A 2 (Task: NC or PC) × 3 (Condition: congruent, neutral or incongruent) × 2 (Group: AMC group or control group) repeated measures ANOVA was carried on accuracy, yielding main effects of Task (*F*_(1,31)_ = 9.17, *p* = 0.005), Congruency (*F*_(2,62)_ = 71.62, *p* < 0.001), as well as a significant interaction effect between Task and Group (*F*_(1,31)_ = 5.14, *p* = 0.031) and a marginal three-way interaction (*F*_(2,62)_ = 2.93, *p* = 0.068).

**Figure 3:**
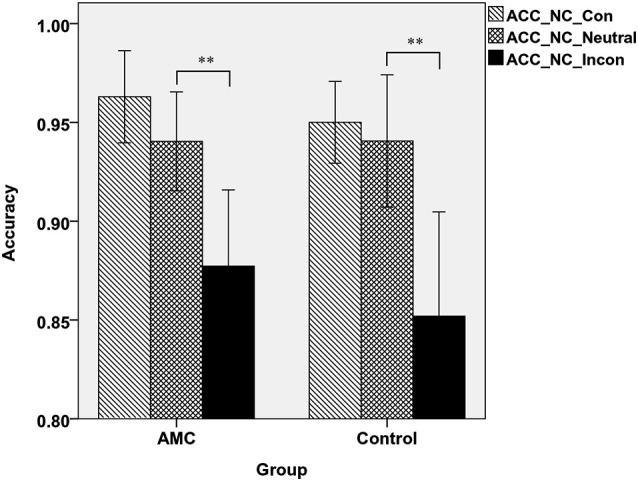
**The accuracies of the two groups in three congruency conditions in NC task**.

A two-way ANOVA analysis revealed that in NC task, there was a main effect of Congruency (*F*_(2,62)_ = 35.97, *p* < 0.001). *Post hoc* analysis revealed that the accuracy in incongruent condition was lower than that in neutral and congruent condition. Specifically, the AMC group showed both interference effect (*p* = 0.001) and a marginal facilitation effect (*p* = 0.077), while the control group only showed interference effect (*p* < 0.001). The mean accuracies of the two groups in NC task were shown in Figure 3.

**Figure 4:**
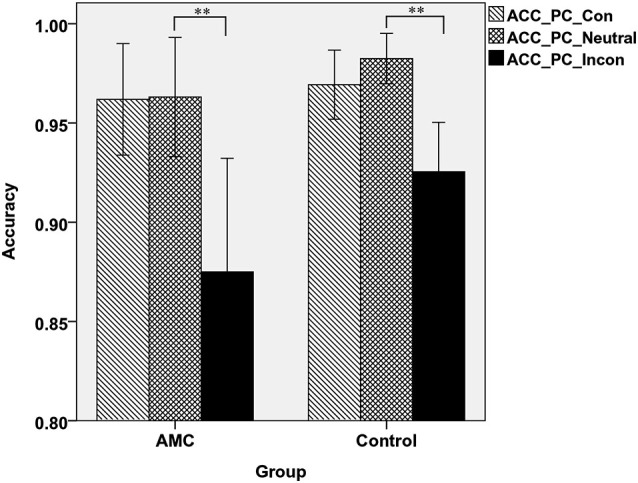
**The accuracies of the two groups in three congruency conditions in PC task**.

Similarly, in PC task there was a main effect of Congruency (*F*_(2,62)_ = 49.84, *p* < 0.001) and an interaction between Congruency and Group (*F*_(2,62)_ = 3.85, *p* = 0.034). A simple effect analysis revealed both groups showed interference effects (both *p* < 0.001) but no facilitation effects. Additionally, the control group had slightly higher accuracy than AMC group in incongruent condition (*p* = 0.066), but not in congruent or neutral condition. The mean accuracies of the two groups in PC task were shown in Figure 4.

#### Sizes of Interference and Facilitation Effects

In line with previous research (Spieler et al., 1996; Girelli et al., 2000), the interference and facilitation effects were quantitatively analyzed. Interference size was calculated as: interference size = (incongruent − neutral)/neutral). Facilitation size was computed as: facilitation size = (neutral − congruent)/neutral.

**Figure 5:**
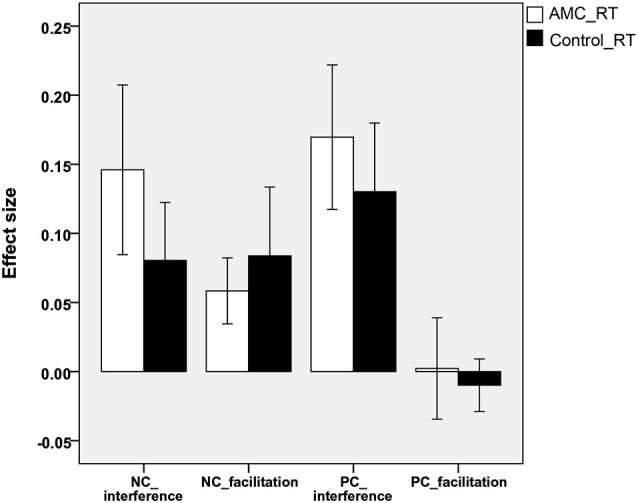
**The interference and facilitation effect size of the two groups in reaction times in NC task**.

**Figure 6:**
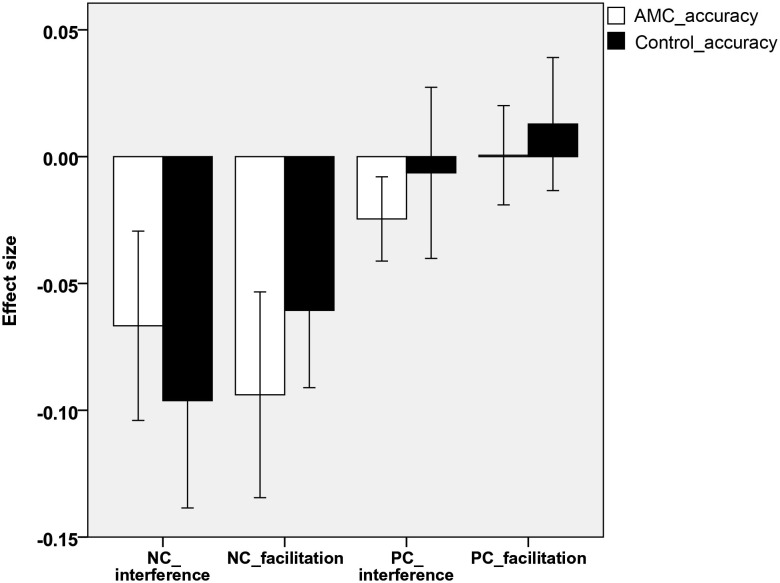
**The interference and facilitation effect size of the two groups in accuracies in PC task**.

Analyses revealed that there were no significant differences between either the sizes of interference or facilitation effects for the two groups. Only the AMC group showed marginally greater interference effect compared to their peers in the NC task (*p* = 0.071). The interference and faciliation effects of two groups in RTs and accuracies were shown in Figures 5, 6.

#### Relative Salience of Numerical Information

To compare the salience of numerical information relative to physical information, we also calculated the RT differences in the neutral condition between NC and PC task, which reflected the speed difference in processing numerical information and physical information (Wang et al., 2013). That is, D = RT(Neutral_NC) − RT(Neutral_PC). Thus, numerical information would be more salient if the D value was smaller.

In the control group, the mean RT in PC task was 232 ms shorter than that in NC task. While in the AMC group, this discrepancy was only 36 ms. The D value in two groups was compared with a *t*-test (*t*_(31)_ = 5.1, *p* < 0.001). Thus, the numerical information was more salient to the AMC group than the control group.

### ERP Results

**Figure 7:**
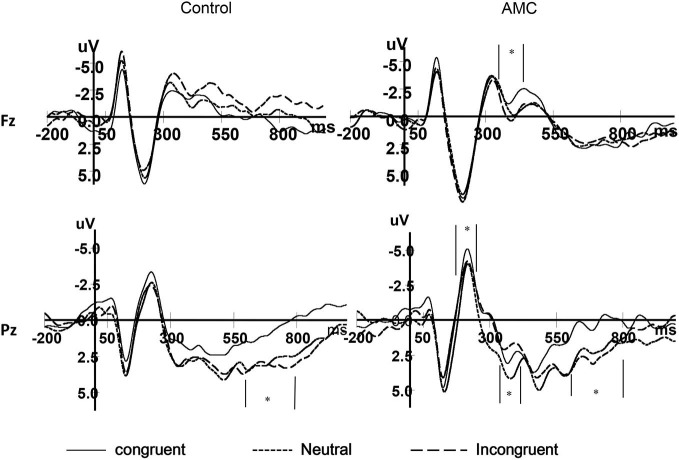
**The grand mean waveforms in three congruency conditions in Fz and Pz site**.

**Figure 8:**
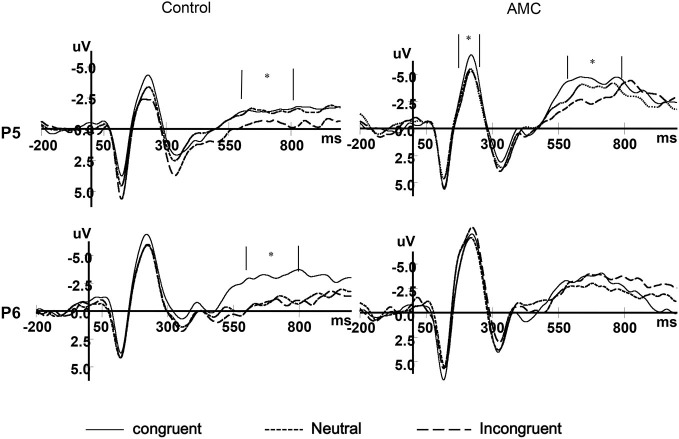
**The grand mean waveforms in three congruency conditions in P5 and P6 site**.

As shown in the behavioral results, the main effect of Group only existed in the NC task. Besides, we were more interested in the numerical processing aspects in this study, thus we only performed the statistics for ERPs during the NC task. The grand mean ERP waveforms were displayed in Figures 7, 8.

#### N1 (170~270 ms)

Based on visual inspection of grand average waveforms and previous EEG studies concerning numerical processing (Dehaene, 1996; Temple and Posner, 1998; Heine et al., 2010), we selected the N1 component. It was mainly distributed in the parieto-occipital area, thus a repeated measures ANOVAs was carried out separately for P5, Pz and P6 sites, with congruency (congruent, neutral, incongruent) as within-subject factors and group (abacus, control) as between-subject factor. The mean amplitues of ERPs in different conditions during 170~270 ms are shown in Table 3.

**Table 3:** **N1 mean amplitudes in each condition (170~270 ms)**.

		P5	P6	PZ
AMC group	Congruent	−4.80 (3.78)	−6.61 (5.81)	−2.94 (2.51)
	Neutral	−3.54 (3.64)	−6.02 (6.60)	−1.81 (2.27)
	Incongruent	−3.70 (3.30)	−7.09 (5.00)	−3.35 (2.92)
Control group	Congruent	−3.24 (3.76)	−5.45 (4.10)	−1.96 (2.62)
	Neutral	−2.59 (3.86)	−4.81 (3.55)	−1.60 (2.20)
	Incongruent	−2.42 (3.96)	−4.84 (2.94)	−1.71 (2.24)

The results of ANOVA revealed significant effects at Pz and P5 sites. No significant results were noted at the P6 site.

At the Pz site, there was a marginal main effect of Congruency (*F*_(2,56)_ = 2.74, *p* = 0.073). Further analysis revealed there was both a facilitation effect (*p* = 0.055) and an interference effect (*p* = 0.011) in the AMC group, but no significant effects in the control group.

At the P5 site, there was a main effect of Congruency (*F*_(2,56)_ = 3.40, *p* = 0.04). Further analysis revealed there was a marginal facilitation effect (*p* = 0.08) in the AMC group but no significant effect in the control group.

#### N2 (370~470 ms)

According to the previous literature and the grand mean waveforms in our results, we found the N2 divergence maily located in the medial frontal and parietal areas. In parallel with behavioral analysis, a repeated measures ANOVA was carried out for Fz and Pz site, with Congruency (congruent, neutral, incongruent) as within-subject factors and Group (AMC, control) as between-subject factor. The mean amplitues of N2 in different conditions are shown in Table 4.

**Table 4:** **N2 mean amplitudes in each condition (370~470 ms)**.

	AMC group			Control group		
	Congruent	Neutral	Incongruent	Congruent	Neutral	Incongruent
Fz	−1.95 (2.81)	−0.68 (4.20)	−0.44 (3.35)	−1.95 (3.15)	−0.94 (3.65)	−1.29 (3.81)
Pz	2.88 (2.49)	3.56 (2.59)	1.75 (3.77)	2.86 (3.70)	2.72 (2.58)	2.25 (2.42)

At the Fz site, there was a main effect of Congruency (*F*_(2,56)_ = 4.26, *p* = 0.024). Further analysis revealed a marginal facilitation effect (*p* = 0.087) in the AMC group, but no significant effect in the control group.

At the Pz site, there was a marginal main effect of Congruency (*F*_(2,56)_ = 3.17, *p* = 0.063). Further analysis revealed an interference effect (*p* = 0.002) in the AMC group, but no significant effect in the control group.

#### LPC (600~800 ms)

According to the previous literature and the grand mean waveforms in our results, we found another divergence in a late positve component (LPC) in the medial parietal area whose polarization was reversed in bilateral parietal areas. The LPC was the late part of the P3b component, and its polarization was reversed in bilateral parietal region. In parallel with behavioral analysis, repeated measures ANOVAs were carried out separately for each site (PZ, P5, P6), with Congruency (congruent, neutral, incongruent) as within-subject factors and Group (AMC, control) as between-subject factor. The mean amplitudes of ERPs in different conditions during 600~800 ms are shown in Table 5.

**Table 5:** **LPC mean amplitudes in each condition (600~800 ms)**.

		P5	P6	PZ
AMC group	Congruent	−4.55 (3.12)	−3.39 (3.43)	0.18 (2.34)
	Neutral	−3.55 (2.32)	−2.63 (4.58)	2.17 (3.29)
	Incongruent	−2.85 (3.20)	−3.63 (3.48)	1.88 (3.00)
Control group	Congruent	−1.84 (2.56)	−3.16 (4.19)	1.07 (2.83)
	Neutral	−1.52 (2.66)	−1.32 (2.99)	2.81 (3.25)
	Incongruent	−1.18 (2.54)	−1.37 (3.39)	2.69 (3.05)

At the Pz site, there was a very significant effect of Congruency (*F*_(2,56)_ = 9.77, *p* < 0.001). Further analysis revealed a facilitation effect (*F*_(1,28)_ = 15.38, *p* = 0.001) and the facilitation effect was significant for both groups (AMC: *p* = 0.008; Control: *p* = 0.012).

At the P5 site, there was a main effect of Group (*F*_(1,28)_ = 6.02, *p* = 0.02) and Congruency (*F*_(2,56)_ = 3.79, *p* = 0.03). No interaction existed between Congruency and Group.

At the P6 site, there was only a marginal main effect of Congruency (*F*_(2,56)_ = 2.67, *p* = 0.085). Further analysis revealed a facilitation effect (*F*_(1,28)_ = 4.06, *p* = 0.05) and the faciliation effect was significant only in the control group (*p* = 0.046).

### Correlation Between Behaviral and ERP Results

As was shown above for LPC, the mean amplitudes recorded over left parietal region also showed main effects of Group and Congruency, which was in parallel with the RT results in the NC task. In order to confirm this assumption, we carried out a Pearson correlation analysis (*n* = 30) in which the two factors were positively correlated to each other in the congruent (*r* = 0.38, *p* = 0.04) and neutral (*r* = 0.47, *p* = 0.009) condition, but not in the incongruent condition (*r* = 0.29, *p* = 0.12).

Compared with neutral and congruent condition, the incongruent condition involved a conflict detection and resolving process, thus we did a partial correlation by controlling for the mean amplitudes of N2 in incongruent condition. Then we found the correlation index became significant (*r* = 0.43, *p* = 0.021).

### Dipole Source Localization

**Figure 9:**
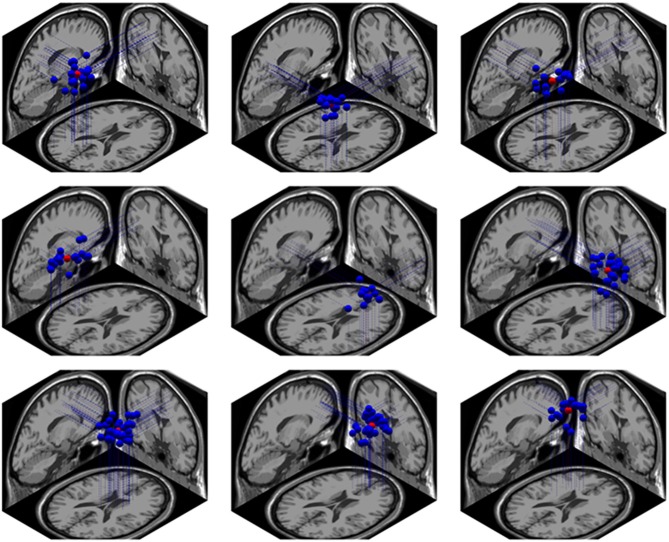
**The brain localization of 9 related dipole sources**. From left to right, the first row represents the left intraparietal sulcus (IPS), right IPS and precuneus; the second row represents the left superior temporal gyrus (STG), right STG and right IFG; the third row represents the cingulate cortex, medial frontal cortex and left MFG.

Using the Dipfit and clustering function in EEGLAB, we found 9 clusters related to the cognitive processing in the NC task. These clusters were localized in bilateral intraparietal sulcus (IPS) and precuneus, right superior frontal gyrus (rSFG), middle frontal gyrus (lMFG), bilateral superior temporal gyrus (STG), medial frontal area, right inferior frontal gyrus (rIFG) and cingulate cortex. The localiztion of these clusters are shown Figure 9.

## Discussion

The current study aimed to examine whether AMC training increases the efficiency of numerical processing using an NSP paradigm. Furthermore, we attempted to examine at what level of the training numerical processing is accelerated by means of EEG recording. To address these issues, we examined interference and facilitation effects in NC and PC tasks for mental abacus and the control group. In parallel, we compared the ERP components of the interference and facilitation effects between the two groups. We also examined the correlations between the mean amplitudes of the LPC component and RTs in the NC task.

### Behavioral Results

The behavioral results can be summarized into four main points. First, consistent with our prediction, the AMC group had shorter RTs than the control group in the NC task. However, no differences were found in the PC task, either in RT or in accuracy between the two groups. Although there is evidence that size and numerical information shared overlapping parietal representation (Pinel et al., 2004), the asymmetry in our results suggests that AMC training enhances the efficiency of processing NC but not physical magnitude. Although, the two dimensions can interact with each other.

Second, no matter whether it was the NC or PC task, both groups showed interference effects in RTs and accuracies. In accordance with previous research using NSP task, congruency effects between numerical and physical information are bidirectional (Henik and Tzelgov, 1982; Tzelgov et al., 1992; Pansky and Algom, 1999; Girelli et al., 2000; Rubinsten et al., 2002; Pinel et al., 2004). This result supports the idea that the two dimensions are processed in parallel and they receive similar involuntary attention when they were the irrelevant information (LaBerge and Samuels, 1974; Henik and Tzelgov, 1982; MacLeod, 1991). From another standpoint, it is possible that visual working memory (VWM) cannot just select an individual feature without processing other basic features from the same objects. Different dimensions of simple features are selected into VWM as integrated objects (Gao et al., 2011), which is consistent with the famous notion of object perception (Kahneman and Henik, 1981; Kahneman et al., 1992).

Third, in the control group, RT was 166 ms shorter (*F*_(1,31)_ = 32.97, *p* < 0.001) in the PC task than in the NC task, which was consistent with several earlier studies (Henik and Tzelgov, 1982: 125 ms; Girelli et al., 2000: 180 ms; Szűcs and Soltész, 2007: 79 ms) suggesting physical magnitude was processed faster than NC. But in the mental abacus group, no speed difference existed between the two tasks. This could be due to the enhancement of numerical information salience in the abacus group. For both groups, facilitation was significant in the NC task but lacked in the PC task, which could also be because of the faster processing speed of physical compared to numerical information. The faster processing of size information could prime the numerical information processing while the slower processing of numerical information could not accelerate the physical size processing (Henik and Tzelgov, 1982; Szűcs and Soltész, 2007).

Our results could also be explained by Logan’s two-process theory. He proposed that the ways of processing information in different tasks was different: the intentional processing is algorithm-based, while the automatic processing is memory-based (Logan, 1980). In the NC task, the goal of the task was number compare, so the way of numerical processing was intentional. But in the PC task, numerical information was irrelevant information and the way of numerical processing became automatic. The AMC group had significantly faster speed than the control group in the NC task, but, the two groups showed no difference either in RT or accuracy in the PC task. This dissociation probably occurred because the AMC group only owned superior processing ability in algorithm-based processing, but not in memory-based processing.

Fourth, there were no group differences in the sizes of facilitation and interference effects in both tasks. This suggests that the two groups had the same efficiency in attending selectively to one feature and filtering out irrelevant features, which is one of the core functions of central executive system of working memory (Baddeley, 1996).

### ERP Results in the NC Task

We demonstrated for the first time the changes in temporal course of numerical processing in the brain induced by long-term training of AMC in children who practiced mental abacus. Congruence effects were found in N1 and N2 components in the mental abacus group but not in the control group in the NC task. Both groups showed congruence effects in LPC. These results suggest that the AMC training accelerated the conflict monitoring process during NC task. Besides, RTs were found positively associated with the LPC component in left parietal region in NC task.

#### N1

N1 amplitude has been found be related to the speed of accessing numerical representation. In studies using a number compare paradigm, N1 displayed a distance effect that reflected the efficiency of accessing NC (Dehaene, 1996; Temple and Posner, 1998; Heine et al., 2010). In our results, the mean amplitude of N1 at central parietal site showed both interference effect and facilitation effect in the abacus group, but not in the control group. A facilitation effect was found in the abacus group and none in the control group over left parietal site. These results suggest an early interaction between physical and numerical dimensions of stimuli processing (Hock and Egeth, 1970) in the abacus group, supporting an overlapping parietal magnitude representations for both physical and numerical information (Pinel et al., 2004). Because our study did not systematically examine the distance between the compared numbers, we cannot know the exact time window when distance effect happened. But the congruency effect that appeared in N1 amplitudes in the abacus group suggests that N1 is not just modulated by numerical distance, but also by the conflict detection process. Although it is earlier than previous studies when conflict detection happens, the scalp distribution of N1 seems to suggest that this process could happen at the sensory and perception stage in the abacus group. This could due to the fact that numerical information is more salient for the abacus group and this conflict detection may only happen when one of the conflict dimensions is numerical information. In other words, this conflict detection may be category specific. So it is possible that the AMC training could accelerate the conflict detection process when processing numerical information.

#### N2

According to previous studies, N2 in correct trials is related to the function of ACC, which plays an important role in the detection of conflict and in alerting higher cognitive systems to resolve this conflict (van Veen and Carter, 2002a). In studies using various tasks, including target search, Go/NoGo and other tasks involving conflict detection, the frontal-central N2 gets larger when the conflict increases (Wijers et al., 1989; Kopp et al., 1996; Smid et al., 1999). For instance, much like in the fMRI study of ACC (Botvinick et al., 1999), the N2 became larger in trials that followed low-conflict trials than in trials that followed high-conflict trials (Wijers et al., 1989). The congruency effect in N2 was also found in studies using classic or numerical Stroop paradigm. Although it appeared later than the N2 in the Go/NoGo task (Liotti et al., 2000; Szűcs et al., 2009), the dipole source localization for the N2 in Stroop task was also in the ACC, consistent with results from neuroimaging studies (Van Veen and Carter, 2002b).

In our results, the N2 amplitude in central frontal site showed a facilitation effect in the abacus group in the NC task. In central parietal site, N2 showed both interference effect and facilitation effect in abacus group, but lacked it in the control group. This group difference could be related to the larger conflict detection in children who practice mental abacus when intentionally processing numerical information and automatically processing physical information. This suggests that the AMC training may strengthen the relationship between symbolic representation and numerical magnitude when they did the comparison and induced larger conflict detection. In other words, numerical information processing may become more automatic in these trained children.

#### LPC

LPC has sometimes been referred to as a late component of the P3b (Finnigan et al., 2002), which originates from activation in the parietal and temporal regions (Polich, 2007). The P3b component is related to a process between perception and response initiation, which associates with attention and appears related to subsequent memory processing (Polich, 2007). Moreover, LPC is related to monitoring whether the stimulus-classification is appropriately translated into action (Verleger et al., 2005). Thus the LPC could be related to the response organization process.

In our results, there were main effects of Group and Congruency over left parietal areas. According to Kok (2001), for tasks that require greater amounts of attentional resources, P3 amplitude is smaller (Polich, 1987; Kok, 2001). In our result, the LPC in Congruent condition showed larger amplitudes than Neutral and Incongruent condtions, which was line with these studies. Moveover, LPC tended to be more negative in the abacus group compared to the control group. Both groups showed faciliation effects over central parietal sites. Only the control group showed facilitation and interference effects over right parietal sites, with amplitudes being more negative in congruent condition than incongruent conditions. These results could be summarized as the abacus group showed a left-lateralized congruence effect while the control group showed congruence effects in bilateral parietal areas in LPC. This suggets that the children who practiced mental abacus could adopt a left-side brain mechanism to resolve conflict and select response while their peers had to employ larger brain area to complete the response organization process.

Interestingly, the left parietal LPC amplitude was positively correlated to RTs in Congruent and Neutral conditions. When controling for the N2 amplitudes in Incongruent condition, the correlation also became significant. Thus, it seems that the higher efficiency of the abacus training group in processing numerical information in NC task could be directly related to the response organization process which was influenced by earlier conflict detection.

#### Dipole Source Localization

fMRI studies have indicated that number was quickly accessed by the dorsal parietal pathway (Cantlon et al., 2006). A recent study combining fMRI and EEG recording found that activation tightly correlated with N1 was observed mainly in a group of parietal areas distributed bilaterally along the IPS, the precuneus as well as in the left middle temporal gyrus and posterior cingulate (Pinel et al., 2001).

Furthermore, Dehaene’s triple-code model predicts three distinct but functionally related systems may be recruited during numerical processing: the horizontal segment of the intraparietal sulcus (HIPS), the left angular gyrus (AG) and the bilateral posterior superior parietal lobule (PSPL). These three systems are respectively related to the internal “number line” (Dehaene and Cohen, 1995; Dehaene, 1996; Chochon et al., 1999; Pesenti et al., 2000; Pinel et al., 2001; Cantlon et al., 2006), the verbal part of the numerical processing system (Snyder et al., 1995; Abdullaev and Posner, 1998; Dehaene et al., 2003), and the visuospatial system (Pesenti et al., 2000; Göbel et al., 2001; Pinel et al., 2001; Dehaene et al., 2003).

Different with adults, young children show more PFC activity when dealing with digital symbols due to lacking full exposure to numbers (Dehaene et al., 2003). As the increase of age and proficiency, this activation shift to posterior parietal and occipito-temporal areas, especially in the left hemisphere (Ansari et al., 2005; Rivera et al., 2005).

In our result, the dipole source of EEG signal were found in bilateral IPS, rSFG, left middle frontal gyrus (lMFG), bilateral STG, medial frontal area, rIFG and cingulate cortex. The activation of IPS, temporal-parietal junction and large frontal areas were highly overlapped with the previous research (Pinel et al., 2001; Ansari et al., 2005; Rivera et al., 2005) and support the triple-code model (Dehaene et al., 2003). Moreover, the activation pattern of cingulate cortex supports the conflict detection theory of ACC (Van Veen and Carter, 2002b).

### Limitations of Study

It should be acknowledged that there were limitations in our study. First, we did not pre-test the two groups for the NSP task, which should provide better understanding of the training from a longitudinal perspective. This concern will be optimized in the further study. Second, our study did not systematically examine the distance between the compared numbers, thus we cannot know the exact time window when distance effect happened. We assumed the N1 component could reflect the numerical magnitude accessing process according to the sound evidence offered by previous studies (Dehaene, 1996; Temple and Posner, 1998; Pinel et al., 2001).

## Conclusion

In conclusion, our results indicate that the abacus training group showed higher efficiency in numerical processing than the control group. The numerical information becomes more salient for the abacus group. Regarding the temporal course of the effect, the abacus group showed earlier congruence effect than the control group in intentional numerical processing. Critically, individual differences in reaction time were positively related to the LPC amplitude in left parietal region in the NC task. Our results suggest that AMC training could strengthen the relationship between symbolic representation and numerical magnitude so that the numerical information processing became more automatic in abacus children and was reflected in the higher numerical processing effiency.

## Conflict of Interest Statement

The authors declare that the research was conducted in the absence of any commercial or financial relationships that could be construed as a potential conflict of interest.

## Footnotes

^1^http://www.sccn.ucsd.edu/eeglab
